# A diagnostic journey started with yellow nail syndrome (YNS) ended with the Kartagener’s syndrome: a rare case report of coexistence of YNS and Kartagener’s syndrome

**DOI:** 10.1097/MS9.0000000000002878

**Published:** 2025-04-02

**Authors:** Mishaim Khan, Noor ul Ain Saleem, Waseem Sajjad, Javed Iqbal

**Affiliations:** a Department of Medicine, FMH College of Medicine and Dentistry, Lahore, Pakistan; b Department of Medicine, King Edward Medical University, Lahore, Pakistan; c Nursing Department Communicable Disease Center, Hamad Medical Corporation, Doha, Qatar

**Keywords:** case report, dermatology, Kartagener’s syndrome, yellow nail syndrome

## Abstract

**Introduction and importance:**

Yellow nail syndrome (YNS) is a rare disorder diagnosed by a triad of yellowish-green discoloration of the nails, respiratory manifestations, and lymphedema. Kartagener’s syndrome (KS) is an autosomal recessive syndrome characterized by chronic sinusitis, bronchiectasis, infertility, and situs inversus. In this case we report the rare coexistence of these solitary occurring disorders.

**Case presentation:**

This report details an interesting case wherein the clinical manifestation of YNS in a 54-year-old male patient not only led to its diagnosis but also revealed the coexistence of another distinctive underlying genetic disorder – KS.

**Clinical discussion:**

KS is an autosomal recessive syndrome characterized by chronic sinusitis, bronchiectasis, infertility, and situs inversus. While KS and YNS are two discernibly unique syndromes, the simultaneous clinical manifestation of their pathologies within a single patient, as presented in our patient, is notably infrequent and rare.

**Conclusion:**

While there is significant documented evidence of YNS co-occurring with only bronchiectasis, we present this case due to the rarity of the amalgamation of YNS with the complete spectrum of pathologies associated with KS. This may help in early diagnosis of either disorder via investigation on the basis of their coexistence as presented in this case and may result in better health outcomes in clinical practice.

## Introduction

Yellow nail syndrome (YNS) is a triad of thickened yellow nails, primary lymphedema, and respiratory manifestations (conventionally including chronic cough, pleural effusion, bronchiectasis, and sinusitis), It is an acquired condition of unknown etiology. It is pertinent to note that YNS is categorized as a syndrome, rather than a disease, often associated with some autoimmune diseases or cancers^[1]^. The first case of YNS was probably reported by Heller in 1927^[2]^. However, Samman & White described the first series of patients who had yellow nails associated with lymphedema in 1964^[3]^. Emerson added pleural effusion to the diagnostic criteria^[4]^. Only two of the three clinical YNS characteristics – yellow nails, respiratory tract involvement, and lymphedema – are crucial for diagnosing YNS. However, it is difficult to call the entity YNS without the nail abnormality^[5]^. While numerous hypotheses in different studies have been proposed to identify the cause of YNS, the definitive pathophysiology of YNS remains unconfirmed.

**HIGHLIGHTS**

- This paper reports a rare case of coexistence of two rare syndromes: yellow nail syndrome and Kartagener’s syndrome.
- It depicts an interesting diagnostic journey where the patient presented with complaints of yellow nails. Upon thorough investigation, it was found that the patient additionally had Kartagener’s syndrome.
- Both of these syndromes are not rare individually, but their coexistence is a very rare.
- This case intricately describes how patient investigation and diagnosis was accomplished with the novel finding of the coexistence of two syndromes.

Kartagener’s syndrome (KS) is an autosomal recessive genetic disorder, characterized by features consisting of a triad of chronic sinusitis, bronchiectasis, and situs inversus. In KS, mutations in the DNAI1 and DNAH5 genes result in impaired ciliary motility, leading to a predisposition for recurrent sinopulmonary infections, infertility, and anomalies in left–right body orientation^[6]^.

Both syndromes exhibit unique and distinctive presentations individually, making their concurrent occurrence an additional layer of exceptional rarity. Based on this rarity we are reporting this case of a 40-year-old male presented with a combination of features of these two rare syndromes. This case report has been reported in line with the SCARE Criteria^[7]^.

## Case presentation

**Figure 1.**
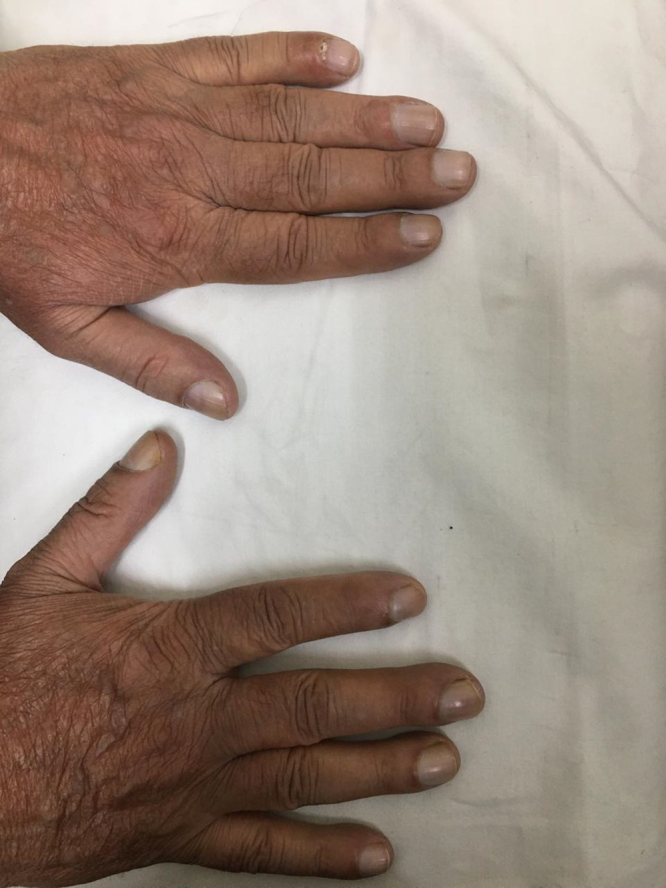
Yellow discoloration of hand nails.

**Figure 2.**
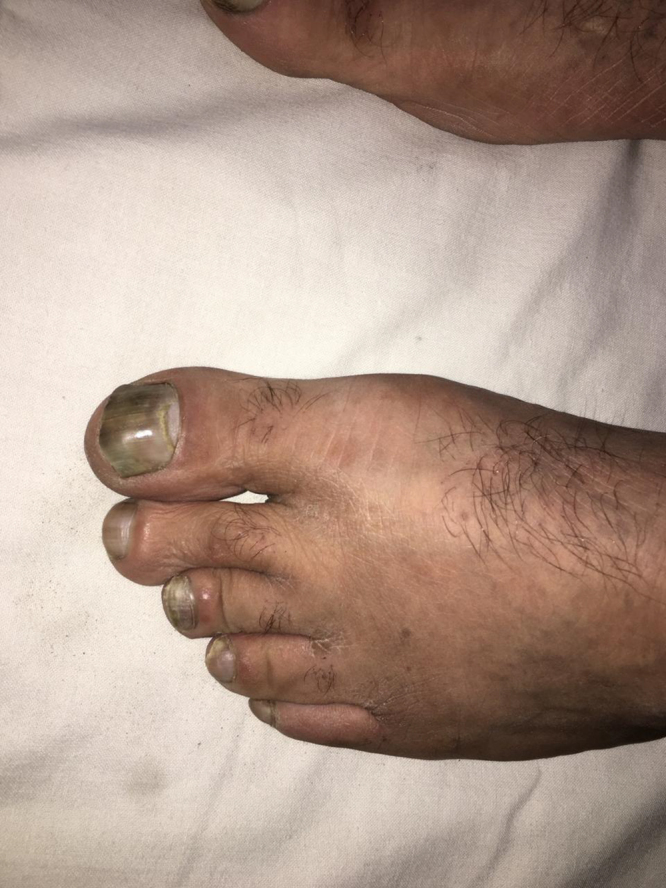
More prominent yellow discoloration of toe nails.

A 40-year-old male presented with yellowish-green discoloration of hands and toenails for 15 years (Figs. 1 and 2). The discoloration was more prominent in the toenails (Fig. 2). Nails were slow growing, thickened, and dystrophic with marked clubbing. He has been applying mixture of topical antifungals and antibacterial ointments off and on, but it didn’t show much improvement in nail discoloration.

On further inquiring, he gave a history of recurrent nasal congestion, occasional discomfort in breathing, and chronic cough sometimes associated with mild-moderate sputum production for 5 years. No significant drug history apart from the application of topical antifungals on nails and a history of intake of some oral antibiotics for sinusitis a few months back. The patient had been in a marital relationship for 13 years but had no offspring in this timeframe although the couple had been trying to conceive in this period.

On physical examination, he was well-nourished, conscious, and oriented. His blood pressure was 110/80 mmHg, pulse rate 85 beats per minute, respiratory rate 20 breaths per minute, and temperature was 37.7°C. His arterial oxygen saturation (SaO2) was 92% on room air.

**Figure 3.**
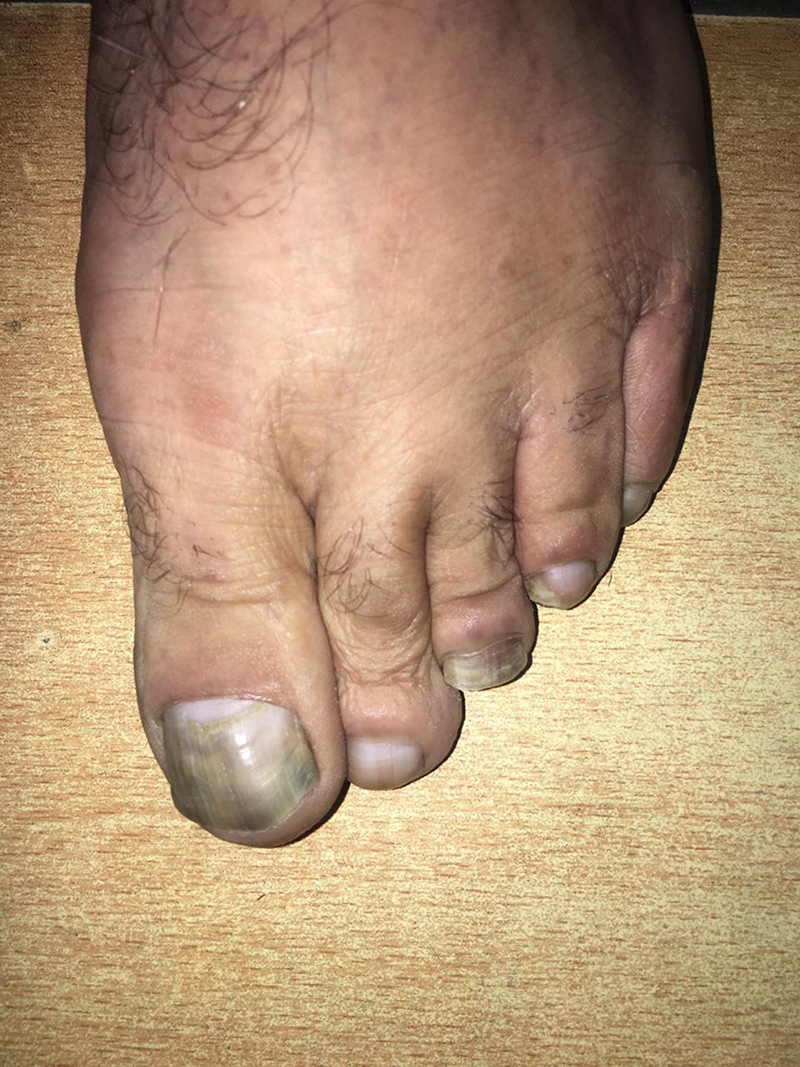
Yellow discoloration and onycholysis.

**Figure 4.**
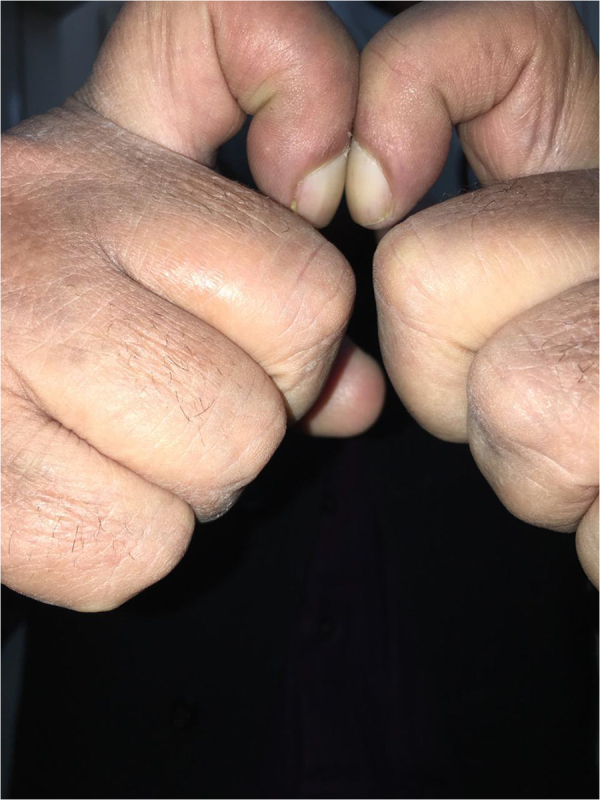
Loss of Schamroth’s window, confirming clubbing.

Nail examination showed yellowish-green discoloration with marked onycholysis (detachment of nail from nail plate) of some nails (Fig. 3). Nails also exhibited thickening and dystrophy. There was a loss of normal angle between nail and nailbed, confirming clubbing of nails (Fig. 4).

Further examination revealed hyperemic conjunctivae, nasal mucosa was also hyperemic with hypertrophied inferior turbinate. A respiratory system examination revealed coarse crackles in both basal lung fields. During the cardiovascular examination, an apex beat was felt on the right fifth intercostal space along the midclavicular line. Heart sounds were particularly noticeable on the right portion of his chest. An abdominal examination revealed a tympanitic percussion note with no sign of fluid collection. Nervous system examination showed no abnormality. No lymphedema was observed at the time of examination.

**Figure 5.**
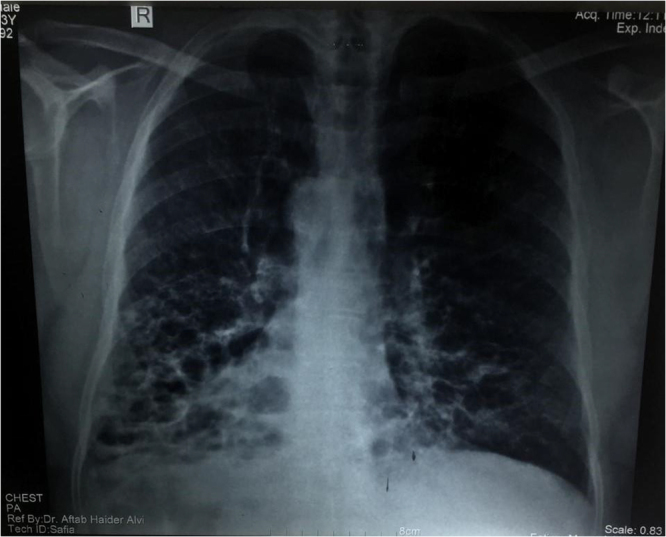
CXR showing right heart and cystic bronchiectic changes.

**Figure 6.**
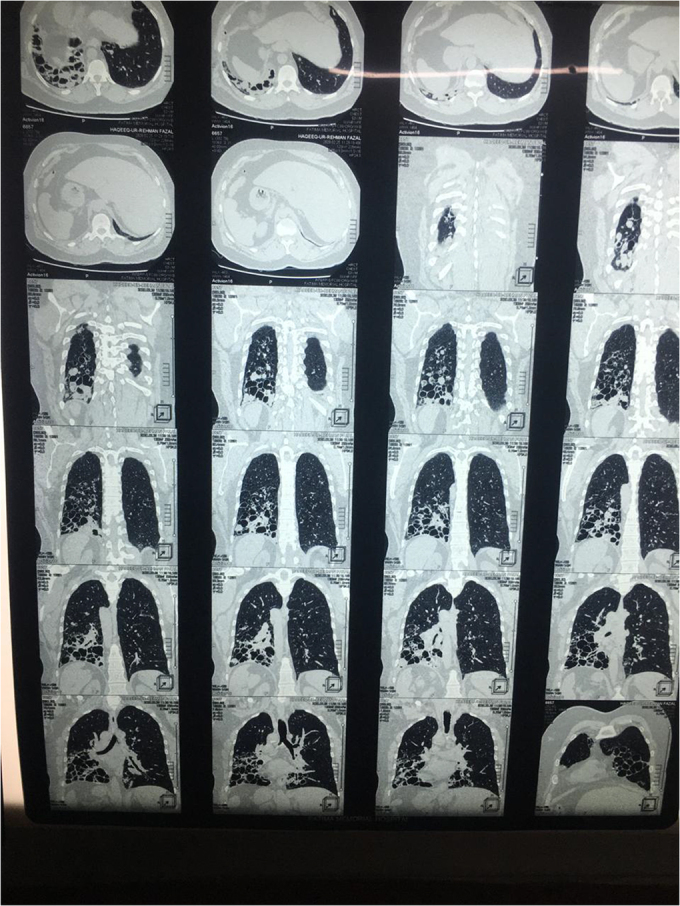
HRCT chest showed a right-sided heart along with cystic bronchiectatic changes in both the right and left lower lobes of the lung.

**Figure 7.**
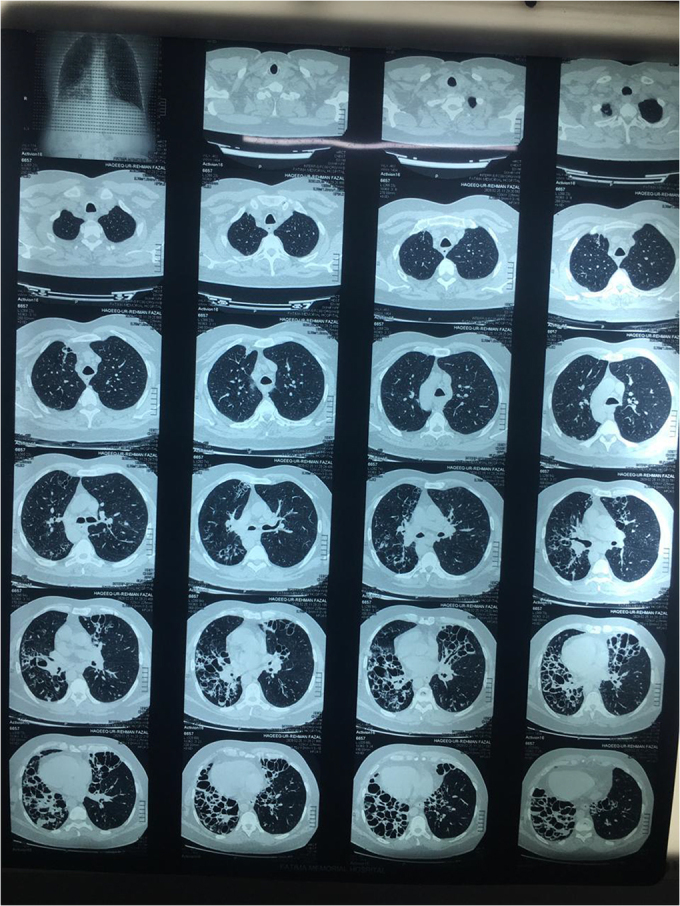
HRCT chest and abdomen showing dextrocardia, left-sided liver, and right-sided spleen, thus indicating situs inversus.

We decided to proceed with radiological investigations of the thorax and abdomen. X-ray and HRCT chest showed a right-sided heart along with cystic bronchiectatic changes in both the right and left lower lobes of the lung, indicating bronchiectasis (Figs. 5–7). It involved both lungs, however dilation of bronchi was prominent in the right lung. Areas of fibrosis and cystic dilation can be seen in the radiograph.

**Figure 8.**
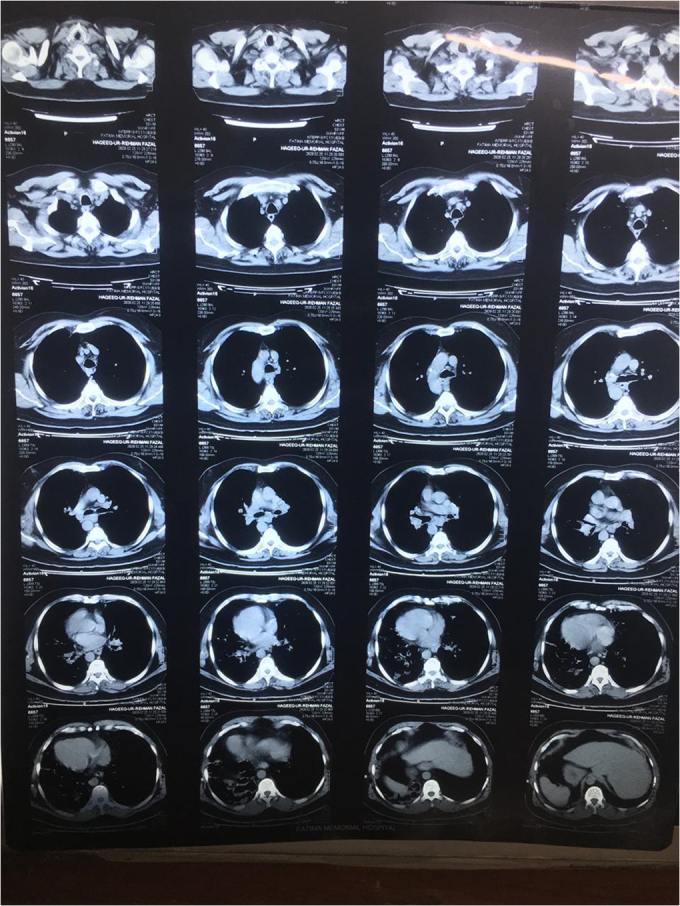
HRCT chest and abdomen showing dextrocardia, left-sided liver, and right-sided spleen, thus indicating situs inversus.

Confirming our examination findings, HRCT chest and abdomen also revealed dextrocardia, left-sided liver, and right-sided spleen, thus indicating situs inversus (Fig. 8). Patient’s overall cardiac health was not affected by his condition. Patient had completely normal lifestyle with his dextrocardia. He had never experienced any dyspnea or chest pain. Heart sounds were normal and no other significant findings were noticed during cardiovascular examination. There was no nail and lung biopsy done.

The dermatology and pulmonology teams were collaboratively engaged, and through a thorough correlation of clinical history, examination, and investigative findings, our patient met the diagnostic criteria for two distinct syndromes – YNS (evidenced by nail pathology and bronchiectasis) and KS (characterized by bronchiectasis, situs inversus, chronic rhinosinusitis, and infertility).

In terms of treatment options, there is presently no specific therapy designated for YNS. However, research indicates favorable responses to a combination of oral fluconazole and oral and topical alpha-tocopherol^[8]^. Zinc supplements may also be considered. In this case, a pulse therapy of itraconazole for three weeks, coupled with oral and topical alphatocopherol (vitamin E), was prescribed. For the concurrent sinusitis and bronchiectasis, a regimen comprising appropriate antibiotics and mucolytics was administered, complemented by chest physiotherapy. Vaccination against influenza and pneumococci was recommended. The patient underwent comprehensive counseling concerning their condition.

Addressing his infertility, detailed history of the patient and gynecological history of his wife were taken. We discussed the relevant investigations and treatment options to evaluate the cause of infertility. But the patient refused to proceed further for infertility analysis. Patient is kept on long term follow up for monitoring of his condition and to prevent any acute exacerbations.

## Discussion

A rare condition known as YNS is typified by thicker, yellow, slow-growing nails, respiratory system involvement, and lymphedema. Discoloration of nails is the main clinical manifestation required to make the diagnosis of YNS. Nail discoloration varies from pale yellow to dark greenish^[9]^. The nail plate becomes thickened, with an enhanced transverse curvature, sometimes associated with a notable hump, very hard nail (scleronychia) and cuticle disappearance^[10]^. YNS most often occurs in adults over 50 years of age, with no sex predominance noted^[11]^. Pediatric forms are very rarely reported^[12]^. However, YNS may be present at birth (congenital) or develop before the age of 10 years^[13]^.

Although there is still no recognized cause for YNS, numerous theories have been proposed. YNS is characterized by lymphedema, which may be the initial symptom in approximately one-third of patients and occurs in 29–80% of documented series^[11,13]^. Manifestations such as lymphedema, pleural effusion (particularly chylothorax), and nail discoloration can be well explained by lymphatic involvement. But it can be difficult to directly connect lymphedema to other related disorders like sinusitis and bronchiectasis, as our patient had. As a result, the pathophysiological function of the lymphatic system in YNS is yet unknown. Lung involvement in YNS, which occurred in 56–71% of the patients, diversely affects the respiratory tract with a variety of clinical manifestations^[13]^. Chronic cough (including bronchiectasis) is the most frequent pulmonary manifestation seen in 56% of YNS patients, with pleural effusions found in 14–46% of the patients^[11-13]^. Recurrent pneumonias occur in 22% of the patients. In addition, YNS patients rarely develop bilateral apical fibrosis, patchy alveolar infiltrates, or cystic lesions.

KS, also known as primary ciliary dyskinesia is a rare, ciliopathic, autosomal recessive genetic disorder that causes a defect in the action of the cilia lining the respiratory tract, genital tract and few other areas of the body. Cilia are also responsible for left to right orientation of the body organs during embryological development.^[14]^. Main genetic mutations involved in pathology are the DNAI1 and DNAH5 genes result in compromised ciliary motility, leading to a predisposition for recurrent sinopulmonary infections, infertility, and anomalies in left–right body orientation^[6]^. As a result, patients commonly appear with chronic recurrent rhinosinusitis, otitis media, pneumonia, bronchiectasis, and a condition called situs inverses^[6,15]^. Although genetic studying on Primary ciliary dyskinesia (PCD) is still an active area of research and needs further clarification, till date, 6 genes have been linked with PCD. Two dynein genes, encoding ODA intermediate chain (DNAI1) and heavy chain (DNAH5) have been seen to be mutated in ~30–38% of the families evaluated. Mutations in other ODA genes (TXNDC3 and DNAI2) have been noted in a small fraction (~2%) of all PCD patients. Recently, mutations have been described in Ktu in ~12% of PCD patients with defects in both the ODA and IDA. Very recently, DNAH11 has been shown to be mutated in a PCD^[16]^.

Genetic testing is not routinely done in most cases due to various reasons. In places where genetic testing is available, it is still challenging due to the extensive genetic heterogeneity and the large size of the disease-causing genes. However, Identification of genes involved in PCD is a very active area of research. However, available genetic testing for PCD can identify approximately one-third of individuals with PCD.

The treatment of KS necessitates a multidisciplinary approach due to the involvement of multiple body systems. The course of treatment is determined by the patient’s clinical presentation. Lymphedema, pulmonary abnormalities and associated symptoms should be managed promptly according to condition of the patient. Any acute exacerbation should be addressed. Symptomatic treatment is typically prescribed for chronic rhinosinusitis. For patients with inadequate symptom control or recurrent exacerbations of bronchiectasis, low-dose antibiotic prophylaxis, such as oral azithromycin, has demonstrated efficacy in alleviating symptoms. Physiotherapy training either alone or in combination with antibiotic prophylaxis, is recommended to assist patients in self-managing chronic expectorations. Immunizations against influenza and pneumococci are strongly recommended. Surgical interventions may prove beneficial for recurrent pleural effusions. Patient is kept on long term follow up for regular monitoring of patient health and to prevent acute exacerbations.

The suggested guidelines for follow-up and prevention of complication include: Pulmonology visits 2–4 times/year; Otolaryngology 1–2 times/year in children, as needed for adults; Audiology at diagnosis and as needed; Reproductive medicine as clinically needed. Long-term surveillance includes chest radiography every 2–4 years, chest CT at least once after age 5–7, airway microbiology cultures 2–4 times/year, non-tuberculosis mycobacterial cultures every 2 years or with unexplained decline, pulmonary function testing 2–4 times/year, and ABPA testing at diagnosis, with new wheezing, or unexplained decline. Preventative therapies: daily airway clearance and nasal sinus lavage, standard vaccinations per local schedule, annual influenza vaccine, 13-valent and 23-valent pneumococcal vaccines per ACIP guidelines, and consider monthly RSV immunoprophylaxis in the first winter^[17]^.

After confirming primary ciliary dyskinesia as the cause of infertility, treatment should begin according to the current available options. Assisted reproductive technology, including in vitro fertilization and ICSI are usually considered for such patients. In vitro fertilization could be a treatment option for patients when sperm motility is retained. However, ICSI is currently the only treatment option for most PCD/KS patients. ICSI overcomes the factors related to impaired motility and bypasses the natural processes for fertilization. Thorough counselling regarding treatment options should be provided to the patient^[18]^.

Mechanism of action of alpha tocopherol in treatment of YNS is not confirmed till date, however therapeutic benefits are thought to be achieved due to its antioxidant properties. It acts either on the abnormal keratinization and arrested nail growth or blocks free-radical-mediated oxidation causing pigment formation (mainly lipofuscin). Recommended dose of oral α-tocopherol is 900 UI daily (600–1200 IU). In a study showing intralesional steroids, such as topical triamcinolone acetonide (5 mg/ml/injection, 0.1–0.2 ml for each affected nail), proposed alone or combined with fluconazole and vitamin E for the treatment of this syndrome showed good response^[19]^.

In a study in which 13 patients were treated with pulse therapy of fluconazole 300 mg weekly and oral vitamin E 1000 IU daily for 18–24 months, 11 patients had complete cure and two patients showed clinical success at 18 months (clinical success was defined as resumption of nail growth by more than 50%)^[20]^.

Differential diagnosis of YNS includes nail discoloration caused by various infectious pathologies which should be ruled out before considering YNS as the definitive diagnosis. This includes nail mycosis caused by candida, aspergillus and some dermatophytes. Pseudomonas aeruginosa, may be responsible for chloronychia (but has green rather than yellow nail discoloration seen in YNS). Other causes include lichen planus, psoriasis or alopecia areata, chronic or acquired paronychia and onychogryphosis^[21]^.

Clinical cases from different parts of the world have been reported with some atypical associations of YNS. This syndrome may be associated with some autoimmune diseases, immunodeficiency disorders, such as common variable immunodeficiency, combined T- and B-cell deficiency, Guillain–Barré syndrome, nephrotic syndrome, Hashimoto’s thyroiditis, severe hypothyroidism or hyperthyroidism, xanthoma granulomatous pyelonephritis, and rheumatoid arthritis. YNS may be associated with some malignant conditions^[1]^.

We found a number of parallels and divergences between our case reports of KS with YNS and other cases that have been previously reported. Research by Nguyen *et al* documented situs inversus and persistent respiratory infections in patients with KS, which is comparable to our situation^[22]^. However, the Nguyen *et al* investigation did not report nail abnormalities, in contrast to our case, who similarly presented with YNS^[23]^. These similarities point to the need for more study to fully comprehend the underlying mechanisms by highlighting the variation in clinical presentations and the possible co-occurrence of YNS in patients with KS.

## Conclusion

YNS and KS have limited evidence of coexisting in a single patient. KS, although recognized as a well-known autosomal recessive disorder with numerous successfully managed clinical cases but in contrast, YNS remains enigmatic in its etiology, despite an increased number of reported cases in recent years. While generally regarded as a sporadic disease, some cases also have been presented with a positive family history of YNS. It is anticipated that as more studies on YNS are documented, its pathophysiology will gain further clarity in the coming years. However, it should be always a fact of consideration that KS and YNS cannot be considered as a diagnostic tool for one another i.e. the presence of one may not confirm or suspect the presence of other until and unless proven otherwise clinically or via investigations.

The patient discussed in this case faced significant challenges due to a missed diagnosis of YNS, leading to use of wrong antibiotics and antifungals for his nail discoloration. This highlights the importance of a meticulous approach to patient history and clinical examination. No single sign should be overlooked; as sometimes apparently minor clues can unveil the diagnosis of an underlying major disease. A thorough correlation of history, examination, and investigations along with critical analysis of all potential associated diseases, is essential in making an accurate diagnosis which may be life saving for a patient.

## Data Availability

None.
